# Rachipagus Parasitic Twin With Epithelialized Myelomeningocele in a Rural Ethiopian Neonate: A Case Report

**DOI:** 10.1002/ccr3.72673

**Published:** 2026-05-11

**Authors:** Biruk Lealem, Elleni Tadesse, Bethelehem Engidawork

**Affiliations:** ^1^ Department of Medicine St. Paul's Hospital Millennium Medical College Addis Ababa Ethiopia; ^2^ Department of Pediatrics and Child Health St. Paul's Hospital Millennium Medical College Addis Ababa Ethiopia

**Keywords:** heteropagus, low resource settings, myelomeningocele, neural tube defect, parasitic twin, rachipagus

## Abstract

Rachipagus parasitic twinning is an exceptionally rare clinical entity resulting from an asymmetric monozygotic twinning process, characterized by a dorsally co‐joined partially formed twin. Co‐ existence of this condition with neural tube defect is uncommon, yet clinically significant. In this paper, we report a neonate who presented with a lumbosacral limb‐like mass and an epithelialized myelomeningocele. Clinical examination showed reduced lower‐limb movements and diminished anal reflex. Investigation with MRI demonstrated posterior vertebral dysraphism, tethered cord, and an externally attached mass containing partially formed osseous elements without visceral organ sharing. Multidisciplinary team planning for complete excision of the parasitic mass with simultaneous myelomeningocele repair and tethered cord management was undertaken at our center, followed by referral to a specialized neurosurgical facility; however, detailed data regarding the management of the neonate after refferal to neurosurgical center could not be obitained due to lack of unified electronic recording system and fragemented refferal pathways and systemic barriers that limited the patient to continue follow up. This case highlights a rare finding that is of significance to clinical and scientific learning, but also the challenges of delivering and sustaining specialized surgical care in low‐resource settings, as well as follow‐ up and documentation of outcomes.

## Key Clinical Message

Co‐occurrence of rachipagus parasitic twin and myelomeningocele in neonates demands prompt MRI for accurate diagnosis of dysraphism and tethered cord and to differentiate it from teratoma. The lesson of this case lies in embryologic insights and early multidisciplinary planning of management with prompt referral, real‐world barriers to pediatric surgery in low‐resource areas that hinder patient follow up and data collection, advocating for improved follow‐up, implementing strategies like telemedicine, integrated extension community health worker involvement in care and documentation.

## Graphical abstract

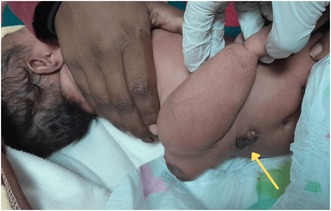

## 1 Introduction

Parasitic twins are rare congenital anomalies, where a partially formed, non‐viable fetus (the parasite) is attached to a fully developed, autosite. Even rarer is the co‐occurrence of this condition with neural‐tube defect [1, 2, 3]. A type of this condition called rachipagus parasitic twinning happens when the attachment occurs to the dorsal spine of the autosite. While heteropagus twins generally occur in approximately 1 in 1,000,000 live births, the specific rachipagus subtype represents a small fraction of these [3, 4, 5, 6, 7].

This condition arises from rare errors during early embryogenesis, primarily explained by two competing theories: incomplete fission, which postulates the mechanism as unsuccessful division of the inner cell mass of a single zygote, and the theory of secondary fusion, which attributes the cause to the result of fusion of initially separate monozygotic embryonic discs at sites of ectodermal deficiency. The co‐occurrence of neural tube defects and rachipagus parasitic twin suggests a widespread disruption during early neurulation. Moreover, the dorsal presentation of a rachipagus twin creates a diagnostic challenge, making it difficult to differentiate it from sacrococcygeal teratoma [3].

There are no clearly identified genetic or environmental risk factors for parasitic twins in general or for rachipagus variety. Existing literature reviews and case series cautiously suggest the link with folic acid deficiency, but this remains a highly speculative and unproven statement [8, 9].

This case report represents the story of a female neonate from rural Ethiopia born with a lumbosacral parasitic twin and an epithelialized myelomeningocele. We have documented the successful diagnostic journey at a tertiary teaching hospital, with a definitive management plan, despite the unfortunate circumstances of being lost to follow‐up after referral. We aim to outline its clinical presentation, rarity, and radiological features; differentiate it from similar conditions like teratomas; and highlight systemic barriers to pediatric surgical care in low‐resource countries, advocating for resilient and better referral and patient tracking systems.

## 2 Case History/Examination

The patient was a 4‐day‐old female neonate born at term (38‐week gestation by early‐trimester ultrasound) from a 28‐year‐old para 2 mother via spontaneous vaginal delivery at home in the rural region of Ethiopia. Her birth weight was 3.07 kg (normal: 2.5–4.0 kg), appropriate for gestational age, with no recorded Apgar scores. The mother reported receiving antenatal care. She claimed that she was not informed of any abnormal findings during her pregnancy follow‐up. Prenatal ultrasound records were not available for review at the time of patient presentation.

The pregnancy was uneventful, with no history of teratogenic exposure, maternal illness, folic acid deficiency, or reported consanguinity, no history of drug use or exposure to radiation during pregnancy, and there was no family history of congenital anomalies, twin pregnancies, or genetic disorders. Within 24 h of birth, the neonate was taken to nearby government health center and 1 day latter to local general hospital in Ethiopia and after careful examination, referral paper was written to our tertiary care teaching hospital for better management of the case with the assessment of term newborn, weight appropriate for gestational age with associated spinal defect and congenital mass (Table 1).

**TABLE 1:** Timeline of patient presentation and diagnostic evaluation.

Day	Event
0 (Birth)	Term home delivery; mass noted; referral to local facility
1–3	Local evaluation; transfer to SPHMMC
4	Admission; assessments and imaging
5–7	Consultations; MRI/ultrasound; neurosurgical referral planned
Post‐7	Discharged; lost to follow‐up

*Note:* Table summarizing the chronological events from birth to referral in a 4‐day‐old female neonate presenting with a lumbosacral rachipagus parasitic twin and associated epithelialized myelomeningocele. The timeline highlights the rapid progression through rural health facilities to tertiary care and the planned multidisciplinary management.

**FIGURE 1:**
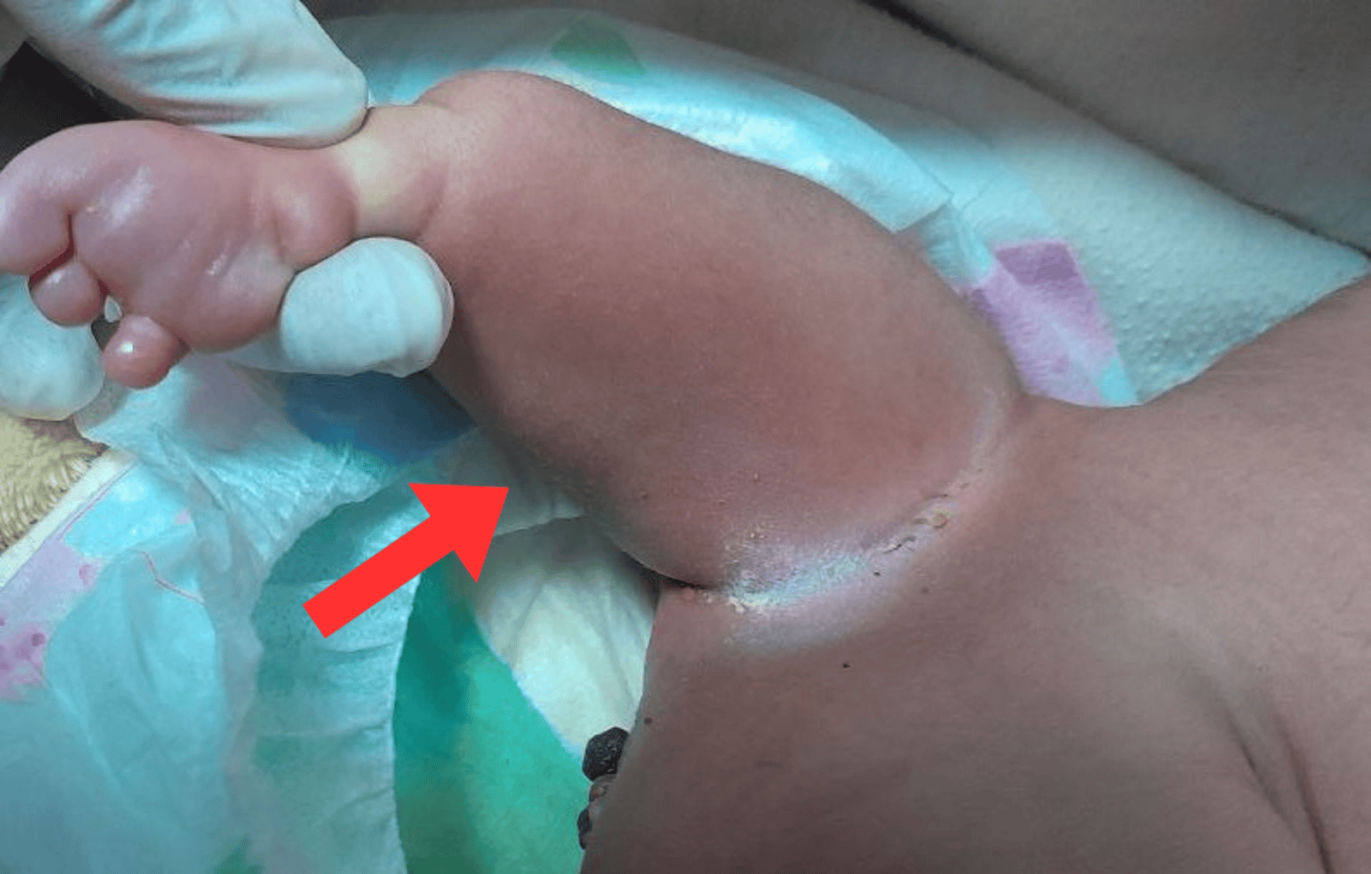
Close‐up picture of distal foot‐like mass indicated by red arrow. Superior view of lumbosacral parasitic limb‐like mass, held for examination. There are several visible non‐motile, soft digit‐like protrusions at the distal end resembling toes, (This organized fetiform morphology supports the diagnosis of rachipagus parasitic twinning).

On admission to our tertiary care hospital, the infant was alert and active. Vital signs were stable: apical heart rate 158 bpm (normal: 120–160 bpm neonatal), respiratory rate 55 bpm (normal: 40–60 breaths/min), temperature 36.8°C (normal: 36.5°C‐37.5°C), and oxygen saturation 95% on room air(normal:95%‐100%). The general examination revealed a pink, non‐icteric neonate with a head circumference of 33.5 cm (normal: 32–36). The anterior fontanel was soft and flat, measuring 2 × 3 cm (normal: 2 × 2 cm to 4 × 4 cm, soft/flat). There was no scalp swelling or suture separation. On musculoskeletal examination, we identified a firm, limb‐like protrusion from the lower back measuring approximately 6 × 8 cm arising from the lumbar region (Figure 1).

**FIGURE 2:**
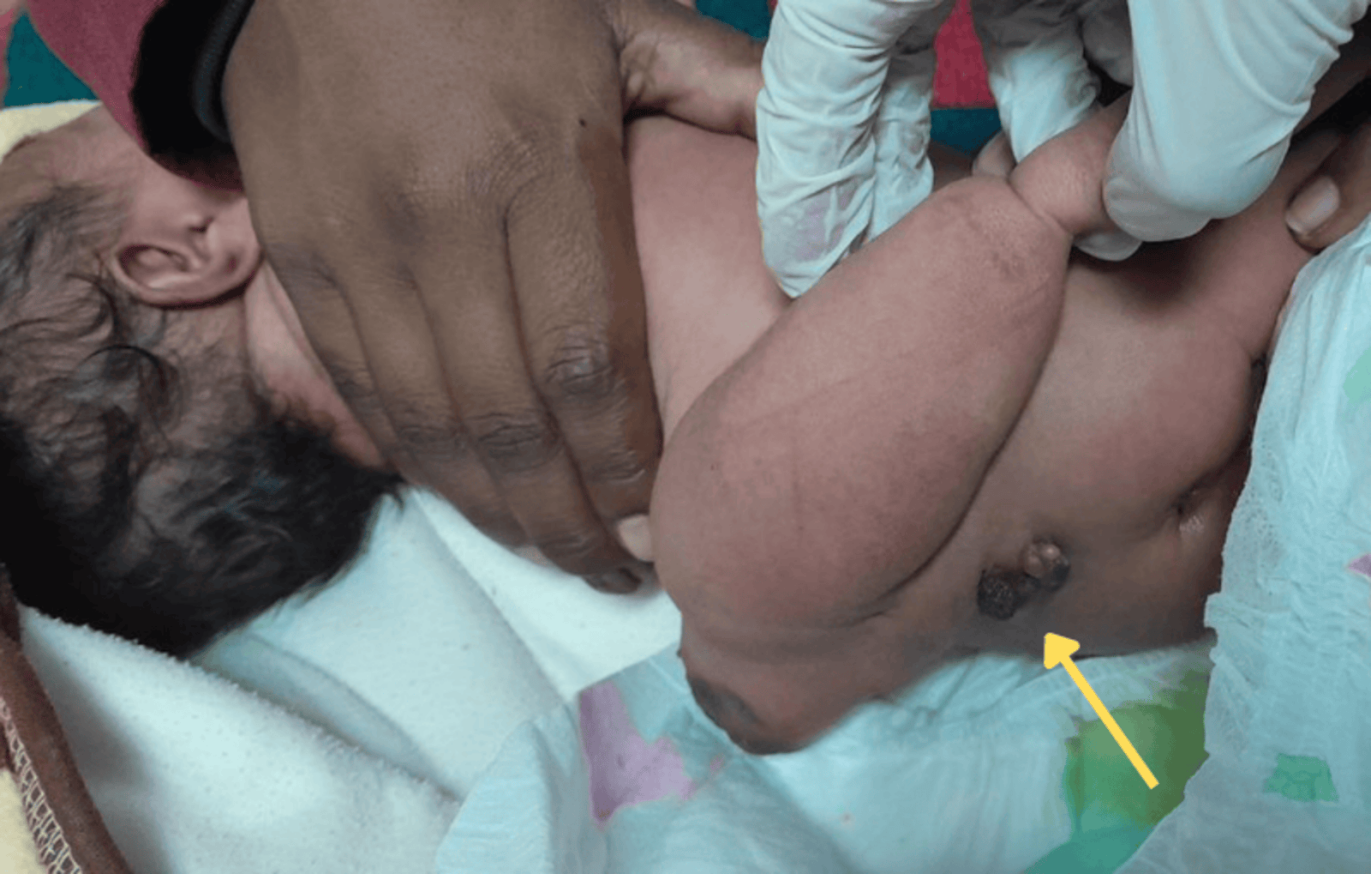
Dorsal attachment of rachipagus twin and spinal defect (shown by yellow arrow) in neonate. Clinical image showing the dorsal attachment of the skin‐covered parasitic twin appendage in the lumbosacral region. The mass exhibits organized morphology with partial limb development. Yellow arrow highlights the adjacent hyperpigmented epithelialized myelomeningocele overlying the site of posterior vertebral dysraphism, as confirmed on subsequent imaging.

The appendage featured a broad base attachment with the back, and its distal end had a morphology resembling a foot, showing several soft, digit‐like projections. Furthermore, the entire mass was covered by intact skin (Figure 2). The limb did not move spontaneously or after stimulation, but the neonate cried when the structure was palpated. At approximately 3 cm below the attachment site, in the midline, there was a focal, darkly hyperpigmented cystic nodule approximately 1 × 1 cm, consistent with epithelialized myelomeningocele, with no active discharge, hair tuft, or drainage noted (Figure 2).

Upon neurologic evaluation, the baby was alert. Upper limb tone, muscle bulk, and spontaneous movements were normal, with intact primitive reflexes. In contrast, lower limb spontaneous movements were present but appeared diminished. Sacral reflex assessment revealed a diminished anal wink reflex and reduced anal tone, with the mother also reporting frequent passage of loose stools and urine dribbling occurring within moments of feeding.

## 3 Differential Diagnosis, Investigations and Treatment

### 3.1 Investigations

Laboratory evaluation results were consistent with physiologic neonatal adaptation: hemoglobin was 20.8 g/dL (normal: 12.7–17.1 g/dL) and hematocrit was 55.0% (normal: 34.4%–48.3%). Total bilirubin was 11.112 mg/dL (normal: 1.3–11.3 mg/dL) and direct bilirubin was 0.260 mg/dL (normal: 0–0.3 mg/dL). Leukocyte analysis revealed a total white blood cell count of 6.26 × 10 “3/mm‐’3 (normal: 3.9–10.1 × 10”3/mm”3) with a differential count showing 57.6% lymphocytes (normal: 17.8%–61.5%) and 26.7% neutrophils (normal: 31.4%–70.5%). Renal and coagulation profiles were normal; no other evidence of infection or organ dysfunction was found.

**FIGURE 3:**
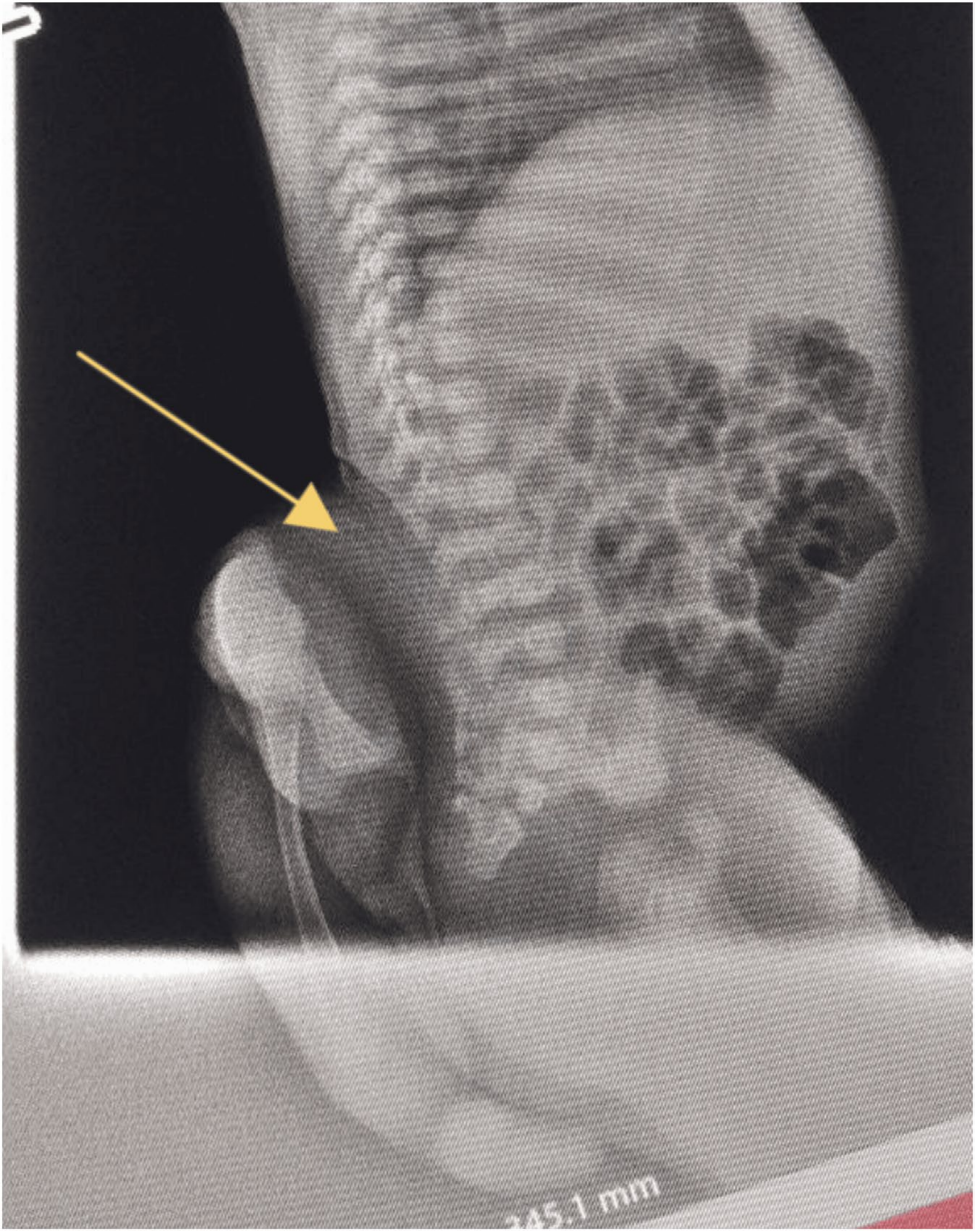
Plain lateral radiograph demonstrating posterior soft tissue mass. Lateral plain radiograph of the lumbosacral spine in a 4‐day‐old female neonate with rachipagus parasitic twinning. The yellow arrow indicates the posterior soft tissue mass containing rudimentary, partially formed osseous elements attached to the site of vertebral dysraphism.

Abdominopelvic ultrasound showed normal visceral morphology, excluding associated internal anomalies Plain lateral lumbosacral radiograph revealed bony dysraphism with a posterior soft tissue mass containing rudimentary skeletal elements, suggesting parasitic twins in contrast to possible complex teratoma (Figure 3).

The MRI shows a complex dual congenital anomaly in the lumbosacral region. There is a large posterior soft tissue mass attached to a posterior bony defect in the lumbar spine associated with the herniated CSF and displaced spinal cord lower tip, features that are strongly linked with a Myelomeningocele (MMC); there was evidence of a Tethered Cord. The mass also contained disorganized, partially formed osseous elements, strongly suggesting a teratoma or parasitic twin. Although the mass appears attached to the site of the spinal defect, there is no major internal organ shared with the host's abdominal cavity. These findings, along with the axial arrangement of tissues, support the diagnosis of parasitic twins.

### 3.2 Differential Diagnosis

The primary differential diagnosis considerations were fetus in fetu, teratoma, and congenital spinal dysraphisms. Fetus in fetu may present as an internal parasitic mass with a vertebral axis but not as dorsal spinal attachment, like wise teratomas consist of disorganized tissue without axial organization and may carry malignant potential.

Additional differential diagnosis that mimics this condition such as polymelia was considered unlikely due to the presence of organized axial skeletal structures rather than isolated supernumerary limb duplication. Smilarly pseudo‐tail was also ruled out because of complex anatomical composition and its epithelialized tissue and limb elements, not matching with benign sacrococcygeal appendages. Other important considerations were cystic hygroma, myelomeningocele, and lipomyelomeningocele.

### 3.3 Therapeutic Intervention

After morning case presentation session, A team comprising neonatologists, pediatricians, and consulting neurosurgeons at St. Paul's Hospital Millennium Medical College reviewed the clinical findings and reached agreement on surgical management after referral to a specialized neurosurgical center for complete excision of the rachipagus parasitic twin mass with simultaneous repair of the epithelialized myelomeningocele and management of the tethered cord.

The neonate was referred to the specialized neurosurgical facility. However, due to the lack of a centralized inter‐institutional electronic medical record system and fragmented referral communication portals, detailed operative notes, intraoperative findings, histopathology results, and the actual postoperative outcome could not be accessed despite repeated contact for follow‐up updates.

While the actual detail of this patient management remains a mystery, In many similar cases, such as this, the standard surgical approach reported by Ethiopian and international literatures involves prone positioning under general anesthesia, making an elliptical skin incision around the base of the parasitic mass then careful dissection along the plane of attachment site to the lumbar spine followed by ligation of vascular pedicles supplying the parasitic structure and finally excision of the mass containing osseous elements along with care to preservation of neuronal structures. The procedure is then completed by making watertight dural closure, and finally layered soft‐tissue reconstruction. Postoperative care usually includes monitoring in the neonatal intensive care unit for possible infection, wound care, giving prophylactic antibiotics, and early physiotherapy to manage preoperative motor deficits [10, 11].

## 4 Discussion

To date, fewer than 80 documented cases of lumbosacral rachipagus twins exist globally, and even fewer in Ethiopia [1, 2, 3, 9, 12, 13]. This condition arises from incomplete fission of a fertilized ovum between days 9–15. The process happens in multiple steps. According to the theory of incomplete fission, the internal zygotic mass undergoes incomplete fission followed by differential growth, where the autosite develops normally while the parasite undergoes ischemic arrest, leaving only a retained vascular pedicle that supports partial organogenesis. Another theory, called secondary fusion, explains the condition that happens due to the fusion of initially separate monozygotic embryonic discs at sites of ectodermal deficiency. These sequences of events then result in a dominant autosite and a growth‐restricted parasite. The histology of the parasite shows mature tissues from all three germ layers arranged along a vertebral axis [3, 4, 5, 14].

Moreover, the location of the attachment sites can vary. In addition, the degree of differentiation may range from limb‐like structures to non‐distinct tissue masses [4, 9, 12, 13]. Furthermore, parasites may be externally or internally positioned and contain varying combinations of skin, bone, cartilage, and neural elements. Rachipagus specifically involves abnormal dorsal axis formation and failed neural tube closure around the time of gastrulation (week 3–4), leading to parasitic attachment along the thoracolumbar spine with frequent split spinal cord [3, 8]. In terms of placental structure, almost always heteropagus twinning results from monozygotic twinning with a shared chorion and amniotic sac [4, 15].

There are no proven genetic or environmental risk factors linked to the development of this condition, partly because of the rarity of this condition and the sporadic nature of its appearance. Reviews cautiously suggest possible associations with folic acid deficiency, particularly in low‐ and middle‐income countries where higher incidences are reported, though this remains unproven and speculative [8, 9].

Definitive diagnosis relies on multimodal imaging to confirm the hallmark features of parasitic twins. Plain radiographs identify bony components and vertebral anomalies, though neonatal films may be limited by incomplete ossification [1, 3, 4, 9, 13]. While computed tomography (CT) provides excellent visualization, its limited availability in low and middle income countries and radiation exposure remains a challenge. On the other hand, Magnetic resonance imaging (MRI) is the gold standard for evaluating soft tissues, spinal involvement, and associated anomalies [3, 13].

The most frequently mentioned differential diagnosis is teratoma. Although similar in gross appearance, teratoma is composed of disorganized tissues without axial orientation, often with immature or malignant components. In contrast, parasitic twins show organized fetiform structures with partial axial development. Other important considerations included fetus in fetu, which is usually situated internally along vertebral axis but lacks dorsal spinal attachment and other disorders such as lipomyelomeningocele. Polymelia (supernumerary limbs without axial skeleton and evidence of true embryologic twining process) and pseudotail (sacral soft tissue overgrowth often indicative of underlying spinal anomaly but lacking specific anatomic organization) were excluded due to the presence of well‐organized vertebral bodies and pelvic bones characteristic of true rachipagus [3, 16].

All differentials were excluded because MRI and clinical findings demonstrated a dorsally attached, externally protruding mass with well‐organized vertebral bodies and pelvic bones directly continuous with the autosite's spine, posterior vertebral dysraphism, and tethered cord—features characteristic of true rachipagus parasitic twinning. In this patient's radiograph, splaying of posterior vertebral elements and MRI‐confirmed vertebral attachment supported the diagnosis of a parasitic mass.

The diagnosis of this case was established by combined clinical and morphologic findings and Image interpretation. Clinically, the presence of limb‐like structures suggests organized body development. In addition, Plain radiography supported this suspicion by showing posterior vertebral dysraphism and associated ossified bones in the mass, while MRI revealed attachment of the mass to the spinal defect, axial arrangement of tissues within the mass, and absence of organ sharing.

The definitive treatment is surgical excision of the parasite, with the goal of preserving autosite body function and neurological integrity [3, 13]. The timing of surgery depends on the anatomy and status of the patient. Neonatal surgery is carried out when attachment is minimal, whereas complex cases may benefit from 6 to 12 weeks of delayed surgery for optimization. and risk reduction [1, 2, 4, 13].

### 4.1 Expected Long‐Term Outcomes

Although actual surgical details and long‐term follow‐up data could not be accessed after referral, previous literatures on similar cases provide a useful prediction for expected outcomes. In cases with minimal visceral sharing with the excised twin and with timely excision, most infants achieve satisfactory neurological recovery, including independent sitting by 9–12 months of age and assisted ambulation. Persistent complications that would occur in this patient, identified from preoperative physical examinations, may include tethered cord syndrome recurrence, hydrocephalus, neurogenic bladder or bowel dysfunction, scoliosis, and lower‐limb orthopedic deformities. Assuming early intervention was done, such cases are generally associated with favorable prognosis whenever postoperative care is within reach of the patient; but, loss to follow‐up precludes assessment of actual neurological, developmental, or quality‐of‐life outcomes in our case [9, 10, 11, 17].

### 4.2 Systemic Barriers to Follow‐Up and Strategies for Care Continuity

This case tries to show the systemic barriers that limit both individual patient care and clinical documentation of rare congenital anomalies in Ethiopia. After the patient's referral to a specialized neurosurgical facility, critical clinical data became inaccessible because of absence of unified or centralized inter‐institutional electronic medical records, further exacerbated by fragmented referral pathways lacking standardized feedback mechanisms, and geographic, financial, and logistical challenges faced by families from remote areas for instance a lot of patients on average travel distances often exceeding 300 km, and spend large sums of money on accommodation. followed by loss of family income earning potential during time spent in healthcare. These structural gaps result in high loss‐to‐follow‐up rates and deprive the global medical community of outcome data that could guide evidence based practices [1, 4, 18, 19].

To address these challenges, we propose the following practical strategies: The first is integration of rare congenital anomaly screening and tracking systems into the existing Health Extension Worker program of the country at community‐level for monitoring and detection of complication and early referral; the second is development of low‐cost telemedicine platforms that would be used in linking rural primary health care facilities with tertiary neurosurgical centers for virtual follow‐up and consultation with senior physicians as well as lab and imaging review; thirdly establishment of a national registry for, not only parasitic twins and neural tube defects but also one that would also encompass other rare congenital malformation that mandate special attention and referral to tertiary hospitals hence also facilitating data sharing and advocacy; and finally policy interventions including arranging transport subsidies and surgical fee waivers for referred patients. Over all such measures, which were also successfully piloted in other chronic‐disease programs in sub‐Saharan Africa, would significantly improve the continuity of care and contribute to the literature database on these rare clinical conditions [20, 21, 22, 23].

## 5 Limitations

Our inability to obtain formal records of operative notes, histopathology results, or long‐term follow up records reflects this limitation and restricts full assessment of treatment outcome, efficiency, and prognosis. This prevents individual patients from receiving optimal longitudinal care but also deprives the global medical community of valuable data on rare congenital anomalies, leaving physicians in a perpetuating knowledge gap that especially affects low‐income countries.

### 5.1 Lessons Learned

This case underscores three learning points: the first is that the diagnosis of a parasitic twin should be entertained as a differential in neonates presenting with a highly differentiated mass associated with spinal defects. Secondly, MRI imaging is critical in reaching a diagnosis and surgical planning. Third, in low‐resource settings, systemic barriers significantly impact continuity of care, outcome documentation, and reporting for rare cases like this.

## 6 Conclusion and Results

### 6.1 Follow‐Up and Outcomes

The neonate's hospital course was smooth with stable vital signs and no acute complications upon referral from the NICU; however the patient was lost to follow‐up after transfer, because the family resided in a remote rural area with limited access to healthcare, accommodation, and transport, as a result postoperative status, histopathologic findings, and long‐term neurologic outcomes could not be assessed.

The journey of this patient encapsulates the profound challenges of travel distance, financial hardship, and centralized care with fragmented referral system that rural people must face to get medical attention. To solve this problem at its root, low‐resource countries must prioritize sustainable options such as establishing national registries for rare conditions, upgrading to low‐cost telemedicine and mobile health platforms for remote monitoring with integrated financial security, such as transport subsidies and fee waivers for referral pathways, and accelerating the introduction of unified electronic medical records across facilities.

### 6.2 Patient Perspective

The mother showed an understanding of the diagnosis and the need for surgical management and was willing to proceed with treatment. However, she complained that the costs associated with her travel, accommodation, and loss of income made continued access to specialized care unbearable. The family's inability to overcome these financial and logistical hurdles ultimately resulted in loss to follow‐up from our center.

## Author Contributions

**Biruk Lealem:** conceptualization, data curation, formal analysis, funding acquisition, investigation, methodology, project administration, resources, software, supervision, validation, visualization, writing – original draft, writing – review and editing. **Elleni Tadesse:** data curation, formal analysis, investigation, methodology, supervision, validation, visualization, writing – review and editing. **Bethelehem Engidawork:** data curation, formal analysis, investigation, methodology, resources, supervision, validation, visualization, writing – review and editing.

## Funding

The authors have nothing to report.

## Ethics Statement

This retrospective case report did not require formal IRB approval per institutional policy, as it relies on written informed consent for publication. The ethical basis for this publication is the written informed consent for publication obtained from the infant's legal guardian, which includes specific consent for the use of clinical images. All patient identifiers have been removed from the manuscript.

## Consent

Written informed consent was obtained from the patient's guardian for publication of this case report and accompanying data. A copy of the consent form is available for review by the Editor upon request.

## Conflicts of Interest

The authors declare no conflicts of interest.

## Data Availability

The data that support the findings of this study are available from the corresponding author upon reasonable request.
